# The Genetics of Life and Death: Virus-Host Interactions Underpinning Resistance to African Swine Fever, a Viral Hemorrhagic Disease

**DOI:** 10.3389/fgene.2019.00402

**Published:** 2019-05-03

**Authors:** Christopher L. Netherton, Samuel Connell, Camilla T. O. Benfield, Linda K. Dixon

**Affiliations:** ^1^The Pirbright Institute, Woking, United Kingdom; ^2^Royal Veterinary College, London, United Kingdom

**Keywords:** African swine fever virus (ASFV), interferon, warthog, *Ornithodoros*, host resistance, host tolerance, viral hemorrhagic fever, DNA virus infection

## Abstract

Pathogen transmission from wildlife hosts to genetically distinct species is a major driver of disease emergence. African swine fever virus (ASFV) persists in sub-Saharan Africa through a sylvatic cycle between warthogs and soft ticks that infest their burrows. The virus does not cause disease in these animals, however transmission of the virus to domestic pigs or wild boar causes a hemorrhagic fever that is invariably fatal. ASFV transmits readily between domestic pigs and causes economic hardship in areas where it is endemic. The virus is also a significant transboundary pathogen that has become established in Eastern Europe, and has recently appeared in China increasing the risk of an introduction of the disease to other pig producing centers. Although a DNA genome mitigates against rapid adaptation of the virus to new hosts, extended epidemics of African swine fever (ASF) can lead to the emergence of viruses with reduced virulence. Attenuation in the field leads to large deletions of genetic material encoding genes involved in modulating host immune responses. Therefore resistance to disease and tolerance of ASFV replication can be dependent on both virus and host factors. Here we describe the different virus-host interfaces and discuss progress toward understanding the genetic determinants of disease outcome after infection with ASFV.

## Introduction

**FIGURE 1:**
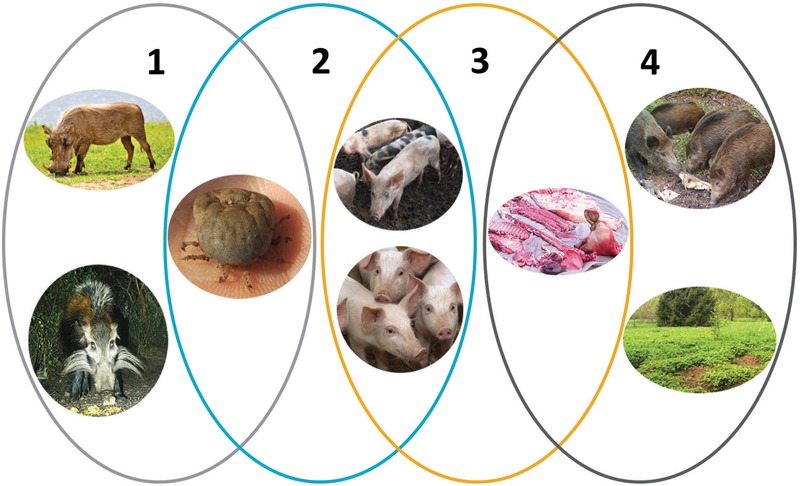
The epidemiologic cycles of African swine fever and main transmission agents. (1) Sylvatic cycle: the common warthog (*Phacochoerus africanus*), bushpig (*Potamochoerus larvatus*), and soft ticks of *Ornithodoros* spp. The role of the bushpig in the sylvatic cycle remains unclear. (2) The tick–pig cycle: soft ticks and domestic pigs (*Sus scrofa domesticus*). (3) The domestic cycle: domestic pigs and pig-derived products (pork, blood, fat, lard, bones, bone marrow, hides). (4) The wild boar–habitat cycle: wild boar (*S. scrofa*), pig-, and wild boar–derived products and carcasses, and the habitat. Reproduced with kind permission from Chenais et al. (2018).

African swine fever virus (ASFV) is present in a stable equilibrium with its wildlife hosts, warthogs and soft ticks of *Ornithodoros* spp., in a unique ecological niche in Eastern and Southern Africa. In these hosts virus can persist over an extended time without causing disease. However, infection of domestic pigs or wild boar with ASFV results in an invariably fatal disease, African swine fever (ASF), which is readily spread between infected pigs or wild boar without the requirement of a tick vector (Figure 1). ASFV can also infect and replicate in bushpigs, but like the warthog these animals do not exhibit clinical signs of disease. Understanding the virus interactions with, and evolution within, these different hosts will help establish the basis for the dramatically varying pathogenesis and potentially unravel the basis for disease resistance of the wild suids in Africa.

ASF was first recognized in the early twentieth century in Kenya as an acute hemorrhagic fever that caused death of most infected domestic pigs (Montgomery, 1921). Early experiments established that warthogs did not show clinical signs of disease but provided a reservoir of infection. ASF was recognized in many Eastern and Southern African countries soon after the initial description and spread further through central and West Africa (Jori et al., 2013; Penrith et al., 2013). From Africa, ASFV expanded into Portugal in 1957 and 1960 and became endemic in the Iberian Peninsula until it was eradicated in the mid-1990s. During this time the disease also became established in Sardinia and sporadic outbreaks were reported in Western Europe, Brazil and the Caribbean. From 1999, with the exception of Sardinia, no further outbreaks of ASF were reported outside of Africa until its appearance in Georgia in the Caucasus region in 2007. Currently ASF is present in sub-Saharan Africa, Sardinia, the Trans Caucasus, the Russian Federation, and Central and Eastern states of the European Union. ASFV continues to spread, with first reports of the disease in China (August 2018), Bulgaria (August 2018), Belgium (September 2018), and Vietnam (February 2019) highlighting the increasing threat of ASF to the global pig industry.

Here we discuss ASFV infection of domestic pigs, wild boar and other wildlife hosts, summarizing current knowledge of how host and viral genetics contribute to pathogenesis and the different disease outcomes seen in different hosts. We also discuss prospects of how these differences might be leveraged to inform breeding or genetic engineering strategies to improve disease resistance in the domestic pig population.

## ASFV Genetics

### ASFV Genetic Variability

African swine fever virus is a large double-stranded DNA virus, which replicates predominantly in the cell cytoplasm and shares a similar replication cycle and genome structure with the poxviruses. However the icosahedral virus morphology differs from the poxviruses and genome sequencing established that ASFV is the only member of a unique virus family, the *Asfarviridae*. Genome sequencing also showed a distant relationship between ASFV and some giant viruses that infect lower eukaryotes, including the Faustovirus, Pacmanvirus and Kamoebaviruses (Reteno et al., 2015; Bajrai et al., 2016; Andreani et al., 2017). Thus these diverse viruses may have shared a common ancestor. ASFV’s genome varies between 170 and 190 kb in length. These gross differences in genome size are predominately due to differences in the copy number of five different multigene families (MGF); for example the copy number of MGF 360 can vary between 11 and 18 in field isolates (Chapman et al., 2008). Promotion of homologous recombination or unequal crossover during DNA replication within infected cells (Rodríguez et al., 1992) is a likely driver of the loss and exchange of genetic material that has been observed in isolates from both ticks and domestic pigs (Dixon and Wilkinson, 1988; Chapman et al., 2011). Interestingly the rapid amplification of individual genes by gene duplication under selection pressure has been observed in poxviruses (Elde et al., 2012) and a similar mechanism may have contributed to differences in the copy number of individual MGFs in ASFV. Paralogs of the MGF genes can be very divergent in sequence indicating evolution over an extended period. This may be related to selection pressure exerted such as altered host tropism. Gene families have evolved in other viruses, for example vaccinia virus encodes a highly divergent family of proteins containing a Bcl-2 protein fold which have different roles in evasion of innate immune responses, including apoptosis and signaling pathways (Graham et al., 2008; Kvansakul et al., 2008; Neidel et al., 2015). Errors in unit genome resolution during the head-to-head DNA replication can also result in sequence transpositions from one genome end to the other as seen in a recent ASFV isolate from northern Estonia that had 14 kb deleted from the left end of the genome and replaced with 7 kb from the right (Zani et al., 2018).

**FIGURE 2:**
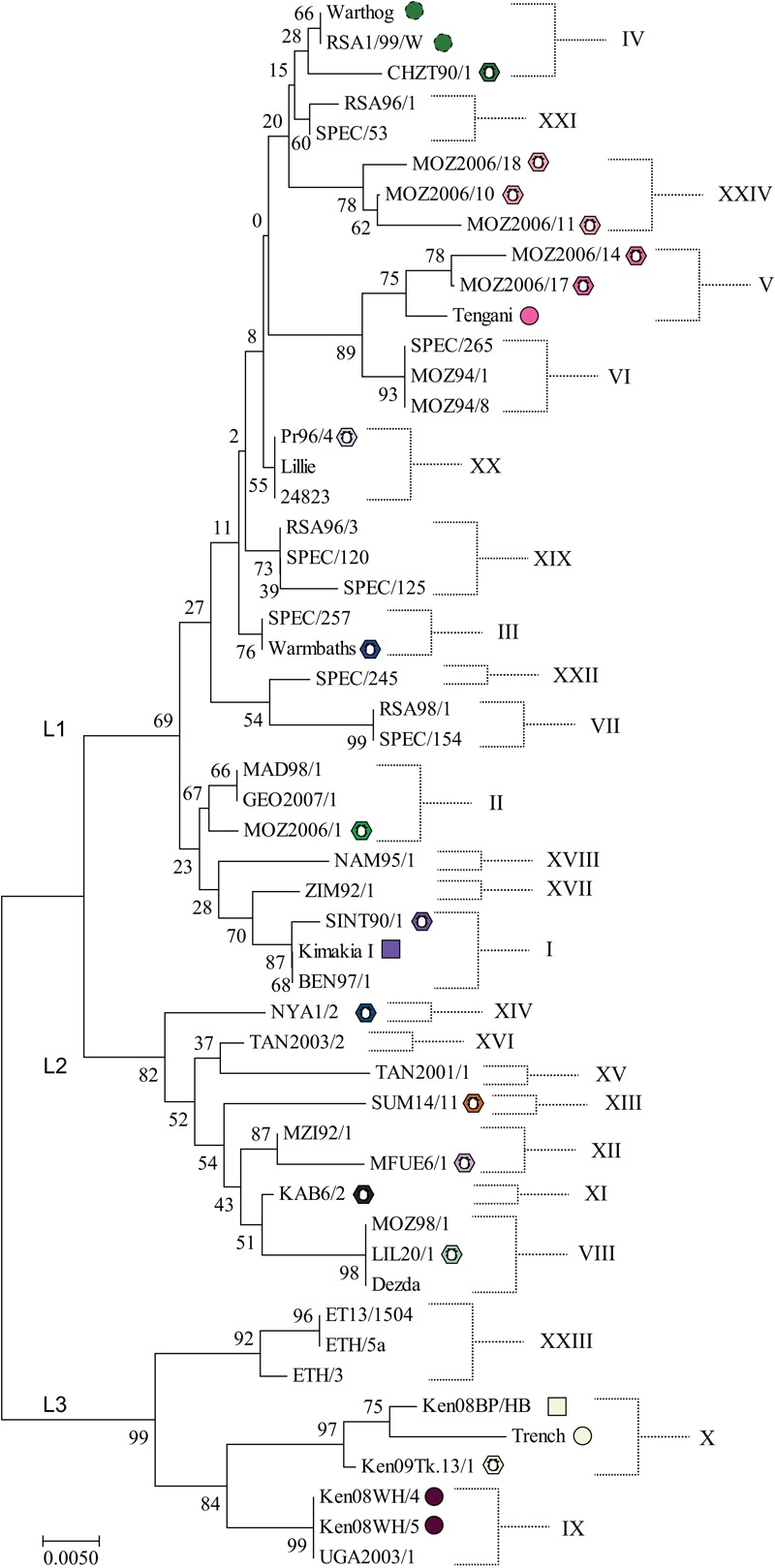
Neighbor-Joining phylogenetic tree of representative ASFV isolates. The evolutionary history was inferred using the Neighbor–Joining method. The optimal tree with the sum of branch length = 0.29203136 is shown. The bootstrap test values (i.e., percentage of replicate trees in which the associated taxa clustered together, 1000 replicates) are shown next to the nodes. The tree is drawn to scale, with branch lengths in the same units as those of the evolutionary distances used to infer the phylogenetic tree. The evolutionary distances were computed using the Kimura 2-parameter method and are in the units of the number of base substitutions per site. The analysis involved 47 nucleotide sequences. All positions containing gaps and missing data were eliminated. There were a total of 399 positions in the final dataset. Evolutionary analyses were conducted in MEGA7 (Kumar et al., 2016). Symbols indicate isolates shown in Figure 3, all other isolates were obtained from domestic pigs. L1, L2, and L3 indicate the lineages identified by Boshoff et al. (2007). Full details of these isolates are provided in Supplementary Table S1.

ASFV isolates have been divided into genotypes based on partial sequencing of the B646L gene which encodes the ASFV major capsid protein. This has defined 24 different genotypes to date (Figure 2; Bastos et al., 2003; Quembo et al., 2018) which fall into three main lineages (Boshoff et al., 2007). However, a limitation of this approach is that the number of nucleotide differences between closely related genotypes can be low. Nevertheless phylogenetic trees constructed from short stretches of conserved genes such as p72 do broadly fit with those generated from concatenated conserved nucleotide or protein sequences (de Villiers et al., 2010). Currently 30 complete genome sequences are available but more than two-thirds of these are of three related genotypes and are therefore clearly not representative of the 24 described genotypes based on p72 sequencing, limiting opportunities to infer evolutionary relationships. Up to 18 genes under positive selection for diversification have been identified by comparing rates of synonymous to non-synonymous substitutions at individual amino acids (de Villiers et al., 2010). These included members of MGF 360 and 505 families, genes involved in modulating host cell functions, several enzymes, the CD2-like and C-type lectin genes and the virus capsid protein chaperone B602L. Drivers for diversification might include immune or host genetic pressure. The major capsid protein did not have any sites under strong selection indicating strong stabilizing selection (de Villiers et al., 2010).

### ASFV Modulation of the Host Response in the Domestic Pig

#### Inhibitors of Type I Interferon

ASFV encodes a number of proteins that inhibit innate immune responses including type I interferon (IFN), the main antiviral response. Stimulation of cellular pattern recognition receptors by an array of pathogen associated molecular patterns induces signaling pathways leading to transcription of type I IFN (Bowie and Unterholzner, 2008; Schoggins et al., 2011; Thompson et al., 2011). The secreted type I IFN activates signaling in infected and bystander cells leading to transcription of over 300 interferon stimulated genes. These include proteins that induce an antiviral state, via blocking the viral replication cycle or activating components of protective innate and adaptive immune responses (Schoggins et al., 2011). For example Mx proteins sequester viral replication factors preventing efficient replication (Netherton et al., 2009) and IFITM proteins restrict virus entry by inhibiting membrane fusion (Benfield et al., 2015).

Although their functional role is currently poorly understood, and they have no obvious similarity to other genes or proteins, there is mounting evidence to suggest that MGF genes may play a role in both host range and subversion of the innate immune system. Sequence analysis indicated that the low virulence isolate OUR T88/3 lacks eight MGF genes (MGF360-10L, 11L, 12L, 13L, 14L, MGF505-1R, 2R, 3R), which are otherwise present in virulent ASFV isolates, suggesting they may play a role in virulence (Chapman et al., 2008; Dixon et al., 2013). Furthermore, levels of IFN in the bloodstream apex prior to the viremic peak, indicating the ability of virulent viruses to endure the host IFN response (Karalyan et al., 2012; Golding et al., 2016). Indeed IFN priming of primary macrophages limited replication of attenuated OURT88/3 but not virulent isolates (Golding et al., 2016).

ASFV lacking these specific MGF genes, including genetically modified virus with the genes in question deleted, induce a stronger innate immune response. Deletion of five MGF360 and three MGF505 from highly virulent Benin 97/1 resulted in attenuation, increased IFNβ production *in vitro* and significantly enhanced protection *in vivo* against challenge with parental virus (Reis et al., 2016). The presence of genes from the MGF360 and MGF505 cluster are directly responsible for supressing IFN responses *in vitro* in cells infected with virulent Pr4 (Afonso et al., 2004) and overcoming IFN mediated inhibition of virus replication (Golding et al., 2016). Further experiments were not able to directly attribute this function to a sole gene or MGF family. A subset of these genes are also important for host range in *Ornithodoros* ticks (Burrage et al., 2004), however the mode of action in the arthropod vector is unknown. Other ASFV genes shown to inhibit type I IFN responses include I329L, an agonist of Toll-like receptor 3 signaling (de Oliveira et al., 2011).

#### Inhibitors of Apoptosis

Induction of apoptosis can limit virus replication and many viruses, including ASFV, encode apoptosis inhibitors (Dixon et al., 2017). These include a Bcl-2 family member A179L, inhibitor of apoptosis member A224L and a C-type lectin protein EP153R (Hurtado et al., 2004). Other ASFV proteins inhibit stress-activated apoptosis (Zhang et al., 2010). The A179L protein has an unusually broad specificity of binding to pro-apoptotic Bcl-2 family BH3 domain-containing proteins (Banjara et al., 2017). This may allow for functionality in both mammalian and *Ornithodoros* hosts.

#### Adhesion Proteins

The ASFV CD2-like protein causes binding of red blood cells to extracellular virions and infected cells. This protein has roles in virus dissemination and persistence in blood in the mammalian host (Borca et al., 1998) as well as facilitating virus uptake into the tick vector (Rowlands et al., 2009). Both of these functions may provide an advantage for virus replication in the tick-warthog cycle.

## Infection and Pathogenesis in Different Host Species

### ASFV in Domestic Pigs

#### Transmission of ASFV to Domestic Pigs and Wild Boar

The emergence of ASFV from its ancient sylvatic ecology in Eastern and Southern Africa, involving warthogs and soft tick vectors of the *Ornithodoros* spp., into domestic pigs and wild boar has resulted in a dramatic change in the pathogenesis of the virus and the mechanisms by which transmission occurs. Transmission by the tick vector is not required in the domestic pig or wild boar cycle and direct transmission between pigs occurs readily in the absence of the tick vector (epidemiological cycles 3 and 4 in Figure 1; Wilkinson, 1984; Guinat et al., 2016). Indeed the ancient sylvatic cycle involving warthogs and ticks has only been described in parts of Eastern and Southern Africa (Jori et al., 2013) meaning that spread through other susceptible populations is unlikely to have placed the same constraints on virus replication.

ASF first spread outside Africa, to Portugal and Spain and from there to a number of other European countries, as well as Brazil and the Caribbean. The disease persisted in the Iberian Peninsula for over three decades, but was eradicated from all of these countries except Sardinia by the mid-1990s. In the Iberian Peninsula ASFV circulated in pigs, wild boar and *Ornithodoros erraticus*, whereas soft ticks did not play a role in Sardinia (Sánchez-Vizcaíno et al., 2015). However, wild boar were not thought to play a significant role in maintaining the virus. In the present epidemic in Russia and Eastern Europe, wild boar have played an important role in spread of disease and maintaining a wildlife reservoir (Abrahantes et al., 2017; Chenais et al., 2018) and there is no evidence of a role for soft ticks. Wild boar show similar clinical signs to domestic pigs and case fatality rates are also close to 100% following infection with highly virulent isolates (Gabriel et al., 2011). Studying the evolution of ASFV clinical forms and associated viral genetic changes during the current epidemic in Europe provides an excellent opportunity to follow the virus adaptation to different hosts.

#### Pathogenesis of ASFV in Domestic Pigs and Wild Boar

Early descriptions of ASF disease in domestic pigs were of an acute hemorrhagic fever causing death of close to 100% of infected pigs (Montgomery, 1921). This is still the predominant disease form reported in both Africa and in Europe (Tauscher et al., 2015). However different disease courses in pigs have been associated with isolates which vary in virulence. Moderately virulent isolates result in death of a lower percentage of animals and a subacute form of the disease. Low virulence isolates may cause few if any deaths and a chronic form of disease characterized by the absence of vascular lesions but signs such as delayed growth, emaciation, joint swelling, skin ulcers and lesions associated with secondary bacterial infection. Moderately virulent and low virulence isolates were described after the introduction of the virus into Spain and Portugal, and similar isolates have now been described from different countries in Africa (Souto et al., 2016) and also in Eastern Europe (Gallardo et al., 2018; Zani et al., 2018). Detection of ASFV specific antibodies in serum from wild boar in Eastern Europe may indicate reduced virulence of circulating isolates, since in acute infections animals die before an antibody response is detected. As yet limited full genome sequences are available for ASFV but reduction in virulence has been associated with genome changes including large deletions and sequence transpositions from one genome end to the other (Zani et al., 2018). Recovered animals may remain persistently infected over extended time periods of weeks or months. Shedding of virus and transmission from recovered animals to in contact animals has been described but it remains unclear whether these carrier animals play an important role in virus spread (Boinas et al., 2004; de Carvalho Ferreira et al., 2012; Gallardo et al., 2015; Petrov et al., 2018). Interestingly ASFV has persisted in Sardinia for 40 years in a pig-pig-wild boar transmission cycle without loss of virulence and with few genetic changes (Granberg et al., 2016; Sanna et al., 2017). Therefore, the main mechanisms of ASFV persistence and transmission in different epidemiological scenarios clearly influence which types of ASFV isolates emerge and become predominant.

#### Influence of Host Genetics on the Outcome of Disease in Pigs

Most reports of ASF disease in domestic pigs or wild boar describe similar acute disease forms with high case fatality in all ages and breeds following infection with highly virulent ASFV isolates (Gabriel et al., 2011; Blome et al., 2013; Nurmoja et al., 2017). However there are also reports indicating differences in susceptibility to disease in some populations or ages of domestic pigs. In one study the percentage of older pigs surviving infection with a moderately virulent isolate was shown to be higher than for younger pigs (Post et al., 2017). In Mozambique, although some pigs were identified that survived infection with a virulent isolate, this apparent resistance was found not to be transmitted to offspring based on results of viral challenge experiments (Penrith et al., 2004). In some regions of Africa apparently healthy pigs have tested positive for virus or had ASFV specific antibodies without showing clinical signs of the disease (Uttenthal et al., 2013; Thomas et al., 2016; Abworo et al., 2017; Kipanyula and Nong’ona, 2017). SNP analysis was used to assess the genetic diversity of two populations of Kenyan pigs and compare them to bushpigs, warthogs, European wild boar as well as four breeds of commercial pigs. Principal component and admixture analyses identified six separate groups, with the two populations of Kenyan pigs forming two distinct groups alongside groups comprised of wild boar, Duroc pigs, African suids or the three other domestic pig breeds (Large white cross, Yorkshire, and Landrace). The failure to resolve bushpigs and warthogs as separate populations was likely due to few markers in the porcine SNP array being amplified in samples from these animals. The Homabay population from Kenya had a local indigenous composition distinct from commercial breeds. In contrast, pigs from Busia and the surrounding area were a non-homogenous admixed population with significant introgression of genes from commercial breeds. Notably a higher percentage of pigs that tested negative for ASFV by PCR had significantly higher local ancestry. Although serology was not performed to prove previous ASFV infection, the study provides some evidence that local ancestry confers a survival advantage against ASFV and a basis to explore genetic determinants underlying resistance to developing disease (Mujibi et al., 2018).

### ASFV in Other Suid Species

The link between outbreaks of ASF and a wildlife reservoir was suspected during the emergence of the disease in the early twentieth century (Montgomery, 1921). Subsequent studies confirmed the isolation of infectious virus from apparently healthy warthogs associated with outbreaks of disease in domestic pigs in both Kenya and South Africa (De Kock et al., 1940; Hammond and Detray, 1955). Infectious virus has been recovered from bushpigs (*Potamochoerus* spp.), warthogs (*Phacochoerus* spp.), *Ornithodoros* ticks and a single giant forest hog (*Hylochoerus meinertzhageni*). The expansion of ASF into South-East Asia raises the possibility of transmission of the virus to other species and genera of suids which have not previously encountered the disease. Warty pigs and bearded pigs (all species of Sus) indigenous to Indonesia and the Philippines would be predicted to suffer similar disease outcomes to domestic pigs and wild boar. However, pygmy hogs (*Porcula salvania*) found in India and babirusa (*Babyrousa* ssp.) from Indonesia are distinct genera (Funk et al., 2007) and their susceptibility to ASFV is unclear; although classical swine fever virus, an RNA virus that causes a disease with similar clinical signs to ASFV, can infect and kill pygmy hogs (Barman et al., 2012). The wild populations of many of these species are of concern with pygmy hogs and Visayan warty pigs (*Sus cebifrons*) considered critically endangered according to the International Union for Conservation of Nature (Narayan et al., 2008; Meijaard et al., 2017). Spill over of ASF into these wild suids could lead to other avenues for exploring disease resistance, but could add an unwelcome pressure on already threatened populations.

#### ASFV in *Potamochoerus* spp.

**FIGURE 3:**
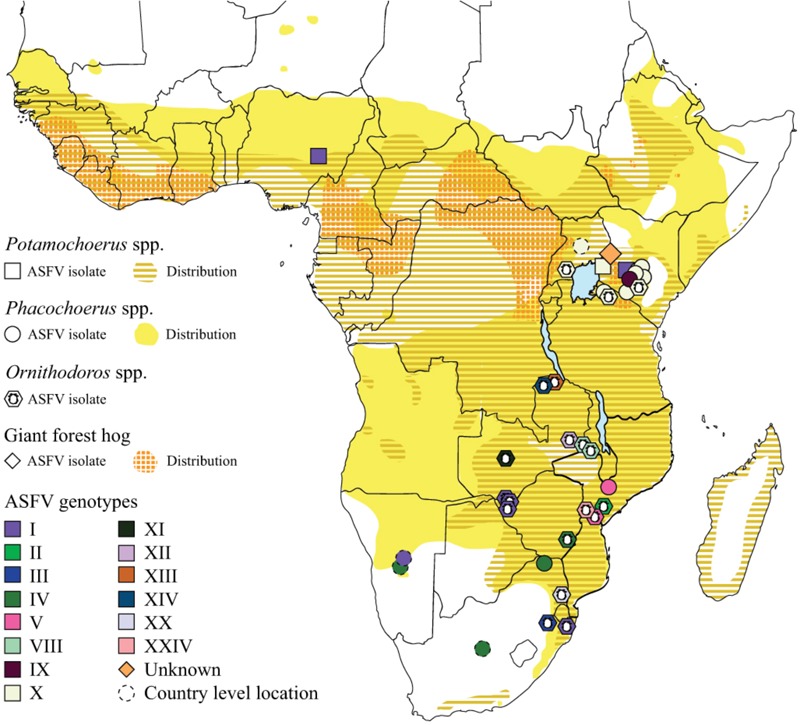
Relationship between distribution of host species and diversity of ASFV. Map of sub-Saharan Africa showing the distribution of bushpigs and red river hogs (*Potamochoerus* spp.), common and desert warthog (*Phacochoerus* spp.) and the giant forest hog. ASFV isolates for which the genotype has been determined are indicated by colored symbols. ASFV isolates from soft ticks (*Ornithodoros moubata* complex) are also indicated. Tick isolates were collected from warthog burrows, with the exception of the two genotype VIII isolates from Malawi and isolates of genotype II and XXIV from Mozambique which were collected from pig holdings. Each symbol indicates a single location which may represent up to 11 separate isolates, full details of these are provided in Supplementary Table S2. The positions of some symbols have been moved to aid clarity where multiple genotypes or hosts have been identified at the same sites.

Bushpigs (*Potamochoerus larvatus*) are distributed throughout Eastern and Southern Africa while red river hogs (*Potamochoerus porcus*) are found in sub-Saharan West and Central Africa. ASFV has been isolated from both bushpigs and red river hogs (Figure 3) and as the two species are closely related and can interbreed we will use bushpigs to refer to all *Potamochoerus* spp. ASFV infection does not induce clinical signs of disease in bushpigs, with virus titres in the blood and tissues 100-fold lower than the 8–9 logs typically seen in domestic pigs (Anderson et al., 1998; Oura et al., 1998a). Virus replication in tissues is also reduced and although extensive B-cell apoptosis in lymph nodes has been observed, this is not as extensive as seen in domestic pigs and other structures are essentially unaffected. Experimentally infected bushpigs clear ASFV from the tissues (Detray, 1963; Anderson et al., 1998) and gain immunity to subsequent rechallenge with homologous virus strains. Bushpigs can transmit virus to feeding ticks and to in-contact pigs. Transmission to pigs depends on the frequency of contacts with domestic pigs and may also be strain specific (Anderson et al., 1998). The role of bushpigs in maintaining a reservoir of virus is unclear since they do not reside in burrows like warthogs and hence are not thought to come into frequent contact with *Ornithodoros* ticks.

#### ASFV in *Phacochoerus* spp.

There are two species of warthog in Africa, the common warthog (*Phacochoerus africanus*) which is distributed throughout sub-Saharan Africa and the desert warthog (*Phacochoerus aethiopicus*) which is restricted to the Horn of Africa and Northern Kenya. We will use the term warthog to refer to the common warthog as to our knowledge ASFV has not been isolated from *Phacochoerus* spp. within the known distribution of the desert warthog, although the recent outbreaks in the Tigray region in northern Ethiopia (Achenbach et al., 2017) suggest that it may only be a matter of time before this occurs. ASFV has been isolated from warthogs across Southern and Eastern Africa (Figure 1), and seropositive animals have also been found in Botswana and Zimbabwe (Jori et al., 2013).

Serological surveys suggest that infection rates in populations of warthogs where ASFV is endemic are typically greater than 80% (Plowright, 1977; Thomson, 1985), although viremia in wild adult warthogs is rare, with infectious virus mostly restricted to lymph nodes. However, wild caught neonatal animals from the Serengeti do exhibit detectable viremia (Plowright, 1977; Thomson, 1985) and experimental infection of naïve young warthogs also yields low viremia for several weeks which may be sufficient to infect ticks (Thomson et al., 1980; Anderson et al., 1998). Therefore it is likely that the warthog-tick sylvatic cycle is in part maintained by ticks transmitting the disease to 3–4 weeks old warthogs that can then transmit the virus to naïve ticks. Interestingly the proportion of ASFV positive ticks in warthog burrows in Western Uganda were found to be very low and the majority of warthogs in this area did not become seropositive until they were 6 months old (Plowright, 1977). In combination with the observation that warthogs in the central Kenyan highlands were seropositive in the absence of ticks, this suggests there a number of different sylvatic cycles capable of maintaining a virus reservoir. Infectious virus persisted in warthog tissues up to 25 weeks post-infection, but is cleared by 56 weeks (Anderson et al., 1998). Field observations have demonstrated persistent infection of warthog tissues (Plowright et al., 1969b; Plowright, 1977). This could be explained by repeated re-infection of warthogs by ticks with the same virus strain. Warthogs probably develop an adaptive immune response to a given ASFV strain, which while insufficient to prevent replication at primary sites of infection can prevent an acute phase and hence virus dissemination into the blood stream.

### ASFV in *Ornithodoros* spp.

African swine fever has been isolated from *Ornithodoros* spp. ticks collected from warthog burrows from Kenya to South Africa (see Figure 1), although the proportion of ticks positive for virus is typically less than 1%. Virus is transmitted sexually and transtadially in ticks (Plowright et al., 1970) and can be isolated from all developmental stages (Plowright et al., 1969a; Quembo et al., 2018). Transovarial transmission of the virus has also been shown in ticks from the *O. moubata* complex. Detailed genetic and morphological analyses of Afrotropical *Ornithodoros* spp. have identified at least four species within each of the *O. savignyi* and *O. moubata* groups, only one of which is not thought to be associated with pigs or warthogs (Bakkes et al., 2018). However, *O. phacochoerus, O. porcinus*, and *O. waterbergensis* are the principal species linked to the sylvatic cycle. Although *O. moubata* spp. are true biological vectors of ASFV virus replication can be deleterious to the tick (Hess et al., 1989) and experimental infection of *Ornithodoros* spp. from the Americas also causes tick mortality (Hess et al., 1987). Therefore, the relative ability of different Afrotropical *Ornithodoros* species to support the replication of different strains of ASFV may be an important aspect of the sylvatic cycle. Genetically related, but distinct strains of ASFV have been identified in ticks from separate warthog burrows within close proximity to each other (Dixon and Wilkinson, 1988; Wilkinson et al., 1988), demonstrating divergent evolution of ASFV within the sylvatic cycle. The sylvatic cycle in Africa provides a reservoir of persistently infected hosts to maintain the infection. In the current situation in Europe and China wild boar and domestic pigs in most cases develop disease with high levels of case fatality. Thus maintaining a virus reservoir requires a readily available pool of susceptible hosts or an effective indirect transmission route.

As yet few virus genes have been identified which confer an advantage for replication in the tick vector (Burrage et al., 2004; Rowlands et al., 2009). A functional genomics approach, involving targeted gene deletions and modifications and testing the effect of these on virus replication in the tick would provide further insights. The lack of a tick cell line susceptible to ASFV infection is a constraint meaning that infections of live ticks is required to achieve this. Further comparative full genome sequencing of virus isolates from tick/warthog and domestic-pig/wild-boar cycles would also help to unravel virus adaptations and selections required for replication in the tick.

## Potential Mechanisms for Host Resistance

Due to the paucity of experimental and genetic data available it is difficult to draw conclusions about why warthogs and bushpigs exhibit limited clinical signs after infection with ASFV when compared to domestic pigs and wild boar. Viral replication is approximately 100-fold lower in bushpigs than in domestic pigs, and replication in warthogs 10-fold less than bushpigs. Comparison of *in vitro* growth curves in macrophages suggest there is no intrinsic difference in the ability of target cells to support the growth of ASFV between the three species (Anderson et al., 1998). It is therefore likely that the innate immune response plays a key role in controlling the levels of virus replication and pathogenesis in different infected hosts. Thus in hosts which do not develop disease the innate immune response may better control virus replication and avoid a pathogenic response. This may involve both viral and host factors. For example virus genetic factors may be less effective in controlling innate responses in the wild African suids compared to the domestic pig or wild boar. Alternatively host genetic factors may reduce over-activation of potentially harmful responses and hence reduced clinical signs may also be due to host tolerance.

ASFV encodes for a diverse combination of genes capable of supressing the induction of type I IFN in domestic pigs. It is tempting to speculate that this functional redundancy of viral IFN inhibitory factors evolved to combat the effect of IFN in the natural host. It would therefore be interesting to compare type I IFN induction and responses in wild African suids compared to domestic pigs and wild boar. Human IFN stimulated genes Mx1 and IFITM (Netherton et al., 2009; Munoz-Moreno et al., 2016) inhibit ASFV replication *in vitro*, however the effect of the *suid* homologs are unknown. Work in our laboratories is currently ongoing to determine the genetic and functional differences between the pig and warthog homologs of these genes.

NK cells are capable of killing virus infected cells, secreting immunomodulatory cytokines and activating dendritic cells, linking with the adaptive immune response. Subclinical infections of domestic pigs with low virulent strains of ASFV and protection in subsequent challenge studies are linked to enhanced NK cell activity (Leitao et al., 2001), so differences in the way these cells respond to ASFV could play a role in the ability of bushpigs and warthogs to control infection.

Interspecies differences in the pathology of ASFV could also be linked to differences in host response to infection. Like many hemorrhagic diseases the pathology of ASFV in domestic pigs has been linked to the overexpression of cytokines such as IFN and tumor necrosis factor alpha (Oura et al., 1998b; Gómez del Moral et al., 1999; Golding et al., 2016). The NF-κB transcription factor controls transcription of both these cytokines and ASFV encodes proteins that can inhibit this pathway (Granja et al., 2006), but these viral NF-κB inhibitors could be less effective in the warthogs and bushpigs compared to domestic pigs or wild boar. Alternatively host transcription factors may be less active in warthogs and bushpigs. For example reporter assays in monkey kidney cells and mouse embryonic fibroblasts show that the RELA subunit of NF-κB from the domestic pig has lower activity after induction by external stimuli than the warthog homolog, but has higher basal activity (Palgrave et al., 2011) and that this difference appears to be due a S531P variant present in the warthog. Genome sequences will help develop additional avenues of research to understand the mechanisms responsible for differences in disease outcomes between domestic pigs, bushpigs and warthogs.

## Conclusion and Future Perspectives

The mechanisms which result in reduced viral replication and lack of disease in African wild suids after ASFV infection are largely unknown. The data so far indicate that this is not due to an intrinsic difference in the ability of the virus to replicate in macrophages from these hosts. A more likely explanation is that the innate immune system of these hosts is better able to control virus replication resulting in a reduced systemic infection and reduced pathogenesis. This may involve a balance between virus and host factors which has evolved over long term infections of these hosts. Sequence information from African wild suids will enable further investigation of the interaction of ASFV with components of the innate immune system compared to domestic pigs and wild boar. A better understanding of ASFV mechanisms of evading host defenses will contribute to this. Of special interest are the functions of the many members of five MGF encoded by ASFV. As is the case in other viruses these may have evolved in the virus genome to modulate the host’s innate immune response.

Genetic modification has been used to generate pigs resistant to porcine respiratory and reproductive syndrome virus (Whitworth et al., 2016; Burkard et al., 2018) or classical swine fever virus (Xie et al., 2018) and therefore could be a viable route to increase resistance to ASFV. Identified warthog or bushpig sequences could be engineered into the pig genome to generate animals in which replication and/or disease burden after ASFV infection is reduced. However, in order to generate a pig that is fully resistant to ASFV infection, as has been accomplished with porcine respiratory and reproductive syndrome virus, a more effective strategy may be to target essential elements of the viral replication cycle such as entry.

Different clinical courses of ASFV infection in pigs have been described, apparently largely due to the virulence of the virus isolates, and sequencing the genomes of isolates of reduced virulence have identified virus genes associated with this phenotype. Targeted gene modifications and deletions and testing of the genetically modified viruses in macrophages and in pigs have contributed to understanding of virulence factors and how the virus modulates host responses. There are no licensed ASFV vaccines available and further research in this area will also contribute to the development of live attenuated vaccines for ASFV.

The issue of whether outcome of ASFV infection in pigs also depends on host genetics has been discussed and considered over a number of years without definite conclusions. Recent studies linking genetics of different pig breeds in Kenya with prevalence of ASFV infection is a promising step forward. Further study of these pigs to confirm that resistance to developing disease after ASFV infection is due to genetic differences rather than hitherto unknown environmental factors could open the possibility of breeding in resistance to the disease. Analysis of other African pig breeds with suspected disease resistance to ASFV may identify additional factors that could be incorporated into such a strategy. Viable bushpig-domestic pig hybrids have been observed in the field and these could open up another avenue of research if these animals were also resistant to disease.

In the longer term a better understanding of ASFV interactions with its different hosts will be not only of great scientific interest but will lead to improved control strategies for this disease and help prevent global spread.

## Author Contributions

CN, SC, CB, and LD wrote individual sections of the manuscript. All authors contributed to the editing and revision of the manuscript as well as read and approved the submitted version.

## Conflict of Interest Statement

The authors declare that the research was conducted in the absence of any commercial or financial relationships that could be construed as a potential conflict of interest.

## Abbreviations

ASF: African swine fever
ASFV: African swine fever virus
IFN: interferon
ISG: interferon stimulated gene
MGF: multigene family
