# Supplementary material for: The Genetics of Life and Death: Virus-Host Interactions Underpinning Resistance to African Swine Fever, a Viral Hemorrhagic Disease
Source: Front Genet. 2019 May 3;10:402. doi: 10.3389/fgene.2019.00402 (PMC6509158; doi:10.3389/fgene.2019.00402)
Supplement: Supplementary file 1 [file Table_1.docx]

**Supplemental Table S1:** Details of ASFV isolates used to construct phylogenetic tree shown in figure 2.

| **Isolate Name** | **Country** | **Location** | **Year** | **Genotype** | **Accession #** | **Reference** |
| --- | --- | --- | --- | --- | --- | --- |
| Benin 1997/1 | Benin | Ladji, Cotonou | 1997 | I | AM712239 | (Chapman et al., 2008) |
| Kimakia I | Kenya | Kimakia | 1961 | I | AY351533 | (Lubisi et al., 2005) |
| SIN T90/1 | Zimbabwe | Sinamatella Camp, North Hwange National Park | 1990 | I | JX235333 | (Takamatsu et al., 2013) |
| GEO 2007/1 | Georgia | Unknown | 2007 | II | AM999764 | (Rowlands et al., 2008) |
| MAD 1/1998 | Madagascar | Unknown | 1998 | II | AF270706 | (Bastos et al., 2003) |
| Moz 1/2006 | Mozambique | Gorongosa National Park | 2006 | II | KY353980 | (Quembo et al., 2018) |
| SPEC/257 | South Africa | Ellisras | 1993 | III | DQ250120 | (Boshoff et al., 2007) |
| Warmbaths | South Africa | Warmbaths | Unknown | III | AY261365 |  |
| Warthog | Namibia | Unknown | Unknown | IV | AY261366 |  |
| RSAW/99/1 | South Africa | Unknown | 1999 | IV | AF449477 | (Bastos et al., 2003) |
| CHZ T90/1 | Zimbabwe | Hippo Valley Estate, Chiredza | 1990 | IV | JX235334 | (Takamatsu et al., 2013) |
| Tengani | Malawi | Tengani | 1960 | V | AF301541 |  |
| Moz 14/2006 | Mozambique | Gorongosa National Park | 2006 | V | KY353993 | (Quembo et al., 2018) |
| Moz 17/2006 | Mozambique | Gorongosa National Park | 2006 | V | KY353996 | (Quembo et al., 2018) |
| MOZ 1/1994 | Mozambique | Maputo | 1994 | VI | AF270711 | (Bastos et al., 2003) |
| MOZ 8/1994 | Mozambique | Manica | 1994 | VI | AF270712 | (Bastos et al., 2003) |
| SPEC/265 | Mozambique | Maputo | 1994 | VI | AF270710 | (Bastos et al., 2003) |
| SPEC/154 | Botswana | Unknown | 1987 | VII | DQ250113 | (Boshoff et al., 2007) |
| RSA 1/98 | South Africa | Potgietersrus | 1998 | VII | AF302818 | (Bastos et al., 2003) |
| Dezda | Malawi | Dezda | 1986 | VIII | AF449479 | (Bastos et al., 2003) |
| LIL 20/1 | Malawi | Kamande, Chalaswa, Michnji | 1983 | VIII | AY261361 |  |
| MOZ 1/1998 | Mozambique | Tete | 1998 | VIII | AF270705 | (Bastos et al., 2003) |
| Ken08WH/4 | Kenya | Kapiti Plains Estates (Machakos) | 2008 | IX | HM745285 | (Gallardo et al., 2011) |
| Ken08WH/5 | Kenya | Kapiti Plains Estates (Machakos) | 2008 | IX | HM745286 | (Gallardo et al., 2011) |
| Uganda 2003/1 | Uganda | Maria Village, Masaka District | 2003 | IX | AY351564 | (Lubisi et al., 2005) |
| Trench | Kenya | Mweiga | 1959 | X | AY351547 | (Lubisi et al., 2005) |
| Ken08BP/HB | Kenya | Nyanza | 2008 | X | JN590911 |  |
| Ken09Tk.13/1 | Kenya | Kapiti Plains Estates (Machakos) | 2009 | X | HM745277 | (Gallardo et al., 2011) |
| KAB 6/2 | Zambia | Kabulushi, Central Kafue National Park | 1983 | XI | AY351522 | (Lubisi et al., 2005) |
| MZI/1/92 | Malawi | Euthini, Mzinda District | 1992 | XII | AY351543 | (Lubisi et al., 2005) |
| MFUE 6/1 | Zambia | Mfue, Luangera National Park | 1982 | XII | AY351561 | (Lubisi et al., 2005) |
| SUM 14/11 | Zambia | Sumbu National Park | 1983 | XIII | AY351542 | (Lubisi et al., 2005) |
| NYA 1/2 | Zambia | Kalumo | 1996 | XIV | AY351555 | (Lubisi et al., 2005) |
| TAN 1/2001 | Tanzania | Dar Es Salaam | 2001 | XV | AY494552 | (Lubisi et al., 2005) |
| TAN 2/2003 | Tanzania | Arusha | 2003 | XVI | AY494551 | (Lubisi et al., 2005) |
| ZIM 1/1992 | Zimbabwe | Gweru Midlands | 1992 | XVII | DQ250119 | (Boshoff et al., 2007) |
| NAM 1/1995 | Namibia | Windhoek | 1995 | XVIII | DQ250122 | (Boshoff et al., 2007) |
| RSA 3/1996 | South Africa | Pienaarsrivier | 1996 | XIX | DQ250127 | (Boshoff et al., 2007) |
| SPEC/120 | South Africa | Potgietersrus | 1987 | XIX | AF302812 | (Boshoff et al., 2007) |
| SPEC/125 | South Africa | Ellisras | 1987 | XIX | DQ250112 | (Boshoff et al., 2007) |
| 24823 | South Africa | Pietersburg | 1975 | XX | DQ250110 | (Boshoff et al., 2007) |
| Lillie | South Africa | Pietersburg Dist, N Transvaal | 1973 | XX | DQ250109 | (Boshoff et al., 2007) |
| Pr 96/4 | South Africa | Pretoriouskop | 1996 | XX | AY261363 | (Boshoff et al., 2007) |
| RSA 1/1996 | South Africa | Gravelotte | 1996 | XXI | DQ250125 | (Boshoff et al., 2007) |
| SPEC/53 | South Africa | Letaba | 1985 | XXI | DQ250111 | (Boshoff et al., 2007) |
| SPEC/245 | South Africa | Louis Trichardt | 1992 | XXII | DQ250117 | (Boshoff et al., 2007) |
| ET13/1504 | Ethiopia | Debre Zeit farm | 2013 | XXIII | KU291454 | (Achenbach et al., 2017) |
| ETH/3 | Ethiopia | Debre Zeit farm | 2011 | XXIII | KT795360 | (Achenbach et al., 2017) |
| ETH/5a | Ethiopia | Bahir Dar farm | 2011 | XXIII | KT795361 | (Achenbach et al., 2017) |
| Moz 10/2006 | Mozambique | Gorongosa National Park | 2006 | XXIV | KY353989 | (Quembo et al., 2018) |
| Moz 11/2006 | Mozambique | Gorongosa National Park | 2006 | XXIV | KY353990 | (Quembo et al., 2018) |
| Moz 18/2006 | Mozambique | Gorongosa National Park | 2006 | XXIV | KY353977 | (Quembo et al., 2018) |

**Supplemental Table S2:** List of ASFV isolated from wild suids and ticks in Africa between 1959 and 2010. Where multiple isolates were obtained from a single site from a single species only one isolate is given. References correspond to the genotyping of a given isolate rather than the original collection, with the exception of the single isolation from a giant forest hog for which the genotype is unknown.

| **Isolate Name** | **Place** | **Host Species** | **Year** | **Genotype** | **Reference** | **Notes** |
| --- | --- | --- | --- | --- | --- | --- |
| Kimakia I | Kimakia, Kenya | Bush pig | 1961 | I | (Lubisi et al., 2005) | 2 isolates of this genotype from this site |
| Ken08WH/4 | Machakos, Kenya | Warthog | 2008 | IX | (Gallardo et al., 2011) | 3 isolates of this genotype from this site |
| Doig | Kiganjo, Kenya | Warthog | 1957 | X | (Lubisi et al., 2005) |  |
| Ken05/Tk1 | Machakos, Kenya | Tick (warthog) | 2005 | X | (Gallardo et al., 2011) | 10 isolates of this genotype from this site |
| Ken08BP/HB | Nyanza, Kenya | Bushpig | 2008 | X | JN590911 |  |
| Ken08Tk.2/1 | Machakos, Kenya | Tick (warthog) | 2008 | X | (Gallardo et al., 2011) | 2 isolates of this genotype from this site |
| Ken09Tk.13/1 | Machakos, Kenya | Tick (warthog) | 2009 | X | (Gallardo et al., 2011) | 8 isolates of this genotype from this site |
| Magadi warthog 1 | Magadi, Kenya | Warthog | 1959 | X | (Lubisi et al., 2005) | 2 isolates of this genotype from this site |
| Trench | Mweiga, Kenya | Warthog | 1959 | X | (Lubisi et al., 2005) |  |
| Davis | Nanyuki, Kenya | Warthog | 1959 | X | (Lubisi et al., 2005) |  |
| Killean I | Nanyuki, Kenya | Warthog | 1959 | X | (Lubisi et al., 2005) | 3 isolates of this genotype from this site |
| Bartlett II | Timau, Kenya | Warthog | 1959 | X | (Lubisi et al., 2005) |  |
|  | Kapenguria, Kenya | Giant Forest Hog | 1965 | Unknown | (Heuschele and Coggins, 1965) |  |
| TENGANI | Tengani, Malawi | Warthog | 1960 | V | (Bastos et al., 2003) |  |
| LIL 20/1 | Chalaswa, Malawi | Tick (pig) | 1983 | VIII | AY261361 |  |
| TIK 82 | Tikoliwe, Malawi | Tick (pig) | 1982 | VIII | KC519628 |  |
| Moz 1/2006 | Gorongosa National Park, Mozambique | Tick (warthog) | 2006 | II | (Quembo et al., 2018) | 11 isolates of this genotype from this site |
| Moz 14/2006 | Gorongosa National Park, Mozambique | Tick (warthog) | 2006 | V | (Quembo et al., 2018) | 3 isolates of this genotype from this site |
| Moz 10/2006 | Gorongosa National Park, Mozambique | Tick (warthog) | 2006 | XXIV | (Quembo et al., 2018) | 3 isolates of this genotype from this site |
| NAM/1/80 | Namibia | Warthog | 1980 | I | (Bastos et al., 2003) |  |
| Warthog | Namibia | Warthog |  | IV | AY261366 |  |
| Bushpig | Plateau State, Nigeria | Bushpig | 2004 | I | (Owolodun et al., 2010) |  |
| Mkuzi/1979 | Mkuzi Game Reserve, South Africa | Tick (warthog) | 1979 | I | AY261362 |  |
| Warmbaths | South Africa | Tick (warthog) |  | III | AY261365 |  |
| RSA W1/99 | Soutpansberg, South Africa | Warthog | 1999 | IV | (Lubisi et al., 2005) |  |
| Pr96/4 | Kruger National Park, South Africa | Tick (warthog) | 1996 | XX | AY261363 |  |
| KIR T89/2 | Kiriwira, Tanzania | Tick (warthog) | 1989 | X | (Lubisi et al., 2005) | 4 isolates of this genotype from this site |
| KIR W89/1 | Kiriwira, Tanzania | Warthog | 1989 | X | (Lubisi et al., 2005) |  |
| KWH/12 | Kiriwira, Tanzania | Warthog | 1968 | X | (Bastos et al., 2003) |  |
| UG10/Tk3.2 | Lake Mburu National Park, Uganda | Tick | 2010 | X | (Achenbach et al., 2017) |  |
| UGA VIR | Uganda | Warthog | 1965 | X | Yu, 1996 |  |
| LIV 10/11 | Livingstone Game Park, Zambia | Tick (warthog) | 1983 | I | (Lubisi et al., 2005) | 8 isolates of this genotype from this site |
| KAB 6/2 | Central Kafue National Park, Zambia | Tick (warthog) | 1983 | XI | (Lubisi et al., 2005) |  |
| MFUE 6/1 | Luangera National Park, Zambia | Tick (warthog) | 1982 | XII | (Lubisi et al., 2005) |  |
| SUM 14/11 | Sumbu National Park, Zambia | Tick (warthog) | 1983 | XIII | (Lubisi et al., 2005) |  |
| NYA 1/2 | Kalumo, Zambia | Tick (warthog) | 1986 | XIV | (Lubisi et al., 2005) |  |
| SINT90/1 | North Hwange National Park, Zimbabwe | Tick (warthog) | 1990 | I | (Takamatsu et al., 2013) |  |
| VICT 90/1 | Victoria Falls, Zimbabwe | Tick (warthog) | 1990 | I | (Bastos et al., 2003) |  |
| CHZT 90/1 | Hippo Valley Estate, Zimbabwe | Tick (warthog) | 1990 | IV | (Takamatsu et al., 2013) |  |

**Supplementary References**

Achenbach, J.E., Gallardo, C., Nieto-Pelegrin, E., Rivera-Arroyo, B., Degefa-Negi, T., Arias, M., et al. (2017). Identification of a New Genotype of African Swine Fever Virus in Domestic Pigs from Ethiopia. *Transbound Emerg Dis* 64(5)**,** 1393-1404. doi: 10.1111/tbed.12511.

Bastos, A.D., Penrith, M.L., Cruciere, C., Edrich, J.L., Hutchings, G., Roger, F., et al. (2003). Genotyping field strains of African swine fever virus by partial p72 gene characterisation. *Arch Virol* 148(4)**,** 693-706. doi: 10.1007/s00705-002-0946-8.

Boshoff, C.I., Bastos, A.D., Gerber, L.J., and Vosloo, W. (2007). Genetic characterisation of African swine fever viruses from outbreaks in southern Africa (1973-1999). *Vet Microbiol* 121(1-2)**,** 45-55. doi: 10.1016/j.vetmic.2006.11.007.

Chapman, D.A., Tcherepanov, V., Upton, C., and Dixon, L.K. (2008). Comparison of the genome sequences of non-pathogenic and pathogenic African swine fever virus isolates. *J Gen Virol* 89(Pt 2)**,** 397-408. doi: 10.1099/vir.0.83343-0.

Gallardo, C., Okoth, E., Pelayo, V., Anchuelo, R., Martin, E., Simon, A., et al. (2011). African swine fever viruses with two different genotypes, both of which occur in domestic pigs, are associated with ticks and adult warthogs, respectively, at a single geographical site. *J Gen Virol* 92(Pt 2)**,** 432-444. doi: 10.1099/vir.0.025874-0.

Heuschele, W.P., and Coggins, L. (1965). Isolation of African swine fever virus from a giant forest hog. *Bull Epizoot Dis Afr* 13(3)**,** 255-256.

Lubisi, B.A., Bastos, A.D., Dwarka, R.M., and Vosloo, W. (2005). Molecular epidemiology of African swine fever in East Africa. *Arch Virol* 150(12)**,** 2439-2452. doi: 10.1007/s00705-005-0602-1.

Owolodun, O.A., Bastos, A.D., Antiabong, J.F., Ogedengbe, M.E., Ekong, P.S., and Yakubu, B. (2010). Molecular characterisation of African swine fever viruses from Nigeria (2003-2006) recovers multiple virus variants and reaffirms CVR epidemiological utility. *Virus Genes* 41(3)**,** 361-368. doi: 10.1007/s11262-009-0444-0.

Quembo, C.J., Jori, F., Vosloo, W., and Heath, L. (2018). Genetic characterization of African swine fever virus isolates from soft ticks at the wildlife/domestic interface in Mozambique and identification of a novel genotype. *Transbound Emerg Dis* 65(2)**,** 420-431. doi: 10.1111/tbed.12700.

Rowlands, R.J., Michaud, V., Heath, L., Hutchings, G., Oura, C., Vosloo, W., et al. (2008). African swine fever virus isolate, Georgia, 2007. *Emerg Infect Dis* 14(12)**,** 1870-1874. doi: 10.3201/eid1412.080591.

Takamatsu, H.H., Denyer, M.S., Lacasta, A., Stirling, C.M., Argilaguet, J.M., Netherton, C.L., et al. (2013). Cellular immunity in ASFV responses. *Virus Res* 173(1)**,** 110-121. doi: 10.1016/j.virusres.2012.11.009.
